# Low-Cost Non-Invasive Microwave Glucose Sensor Based on Dual Complementary Split-Ring Resonator

**DOI:** 10.3390/s26072056

**Published:** 2026-03-25

**Authors:** Guodi Xu, Zhiliang Kang, Xing Feng, Minqiang Li

**Affiliations:** 1 College of Mechanical and Electrical Engineering, Sichuan Agricultural University, Ya’an 625014, China; 13408408533@163.com; 2 College of Science, Sichuan Agricultural University, Ya’an 625014, China; 3 School of Electronic Engineering, Chengdu Technological University, Chengdu 610031, China; minqiangli@163.com

**Keywords:** dual complementary split-ring resonator (DS-CSRR), glucose concentration, non-invasive, microwave sensor

## Abstract

**Highlights:**

A low-cost, non-invasive microwave glucose sensor based on a Dual Complementary Split-Ring Resonator (DS-CSRR) is proposed. The introduction of two U-shaped slots into the structure significantly enhances the resonator’s quality factor (Q-factor) and electric-field concentration. Operating at 3.3 GHz, the sensor achieves a sensitivity of 1.95 kHz/(mg·dL^−1^) for glucose-solution detection, offering a compact, easy-to-fabricate, and inexpensive solution for non-invasive blood-glucose monitoring.

**What are the main findings?**

- The modified Dual Complementary Split-Ring Resonator (DS-CSRR) design increases the quality factor (Q) to 130 compared with the conventional DS-CSRR 65 under no-load conditions, indicating a substantial improvement in resonant sharpness and frequency resolution.
- Experimental measurements show a linear frequency shift with different glucose concentrations, yielding a sensitivity of 1.95 kHz/(mg·dL^−1^), which confirms the sensor’s high responsiveness to glucose level variations.

**What are the implications of the main findings?**

- By employing a low-cost FR4 substrate and standard PCB fabrication process, the sensor offers a non-invasive and reagent-free approach for glucose detection, paving the way for affordable, continuous monitoring of personal glucose meters.
- The structural optimization—achieving both a higher Q factor and stronger field confinement within a small footprint—offers a useful design strategy for developing miniaturized, high-sensitivity microwave biosensors.

**Abstract:**

Rapid and real-time monitoring of blood glucose concentration is critical for the diagnosis and management of diabetes, while conventional invasive detection methods suffer from inconvenience and discomfort, making non-invasive detection a research hotspot. In this study, a dual complementary split-ring resonator (DS-CSRR) operating at 3.3 GHz was designed and fabricated for non-invasive glucose concentration detection, aiming to address the problems of low sensitivity and large size of existing microwave glucose sensors. The sensor was fabricated on a low-cost FR4 dielectric substrate with dimensions of 20 × 30 × 0.8 mm^3^, and two U-shaped slots were incorporated into the traditional DS-CSRR structure to realize cross-polarization excitation. This design not only enhances the interaction between the electric field and glucose solution but also optimizes the quality factor (Q) and electric field distribution of the resonator without changing the overall size. Compared with the traditional DS-CSRR, the Q factor of the modified structure is increased to 130 under no-load conditions. The transmission coefficient Signal Port 2 to Port 1 (S21) of the sensor loaded with glucose solutions of different concentrations was measured using a vector network analyzer (VNA). The experimental results show a good linear frequency shift with the increase in glucose concentration, with a measured sensitivity of 1.95 kHz/(mg·dL^−1^). In addition, the sensor is characterized by miniaturization, low cost and easy fabrication due to the adoption of standard PCB fabrication processes. This study successfully demonstrates a non-invasive microwave sensor with high sensitivity for glucose concentration detection, which has promising application potential in personal continuous glucose monitoring, and also provides a useful design strategy for the development of miniaturized high-sensitivity microwave biosensors.

## 1. Introduction

Diabetes has become a major global health challenge, with 537 million adults affected worldwide in 2021—approximately one-tenth of the adult population—and projections suggesting a rise to 784 million by 2045 [1]. Primarily characterized by glucose metabolism disorders and hyperglycemia, diabetes results from insulin deficiency or resistance and poses significant risks due to its chronic nature and severe complications [2]. Effective management of the disease hinges on the frequent and accurate monitoring of blood glucose levels. Conventional invasive methods, though reliable, are inconvenient and carry discomfort, highlighting the pressing need for non-invasive, real-time monitoring solutions. Microwave-based sensors offer a promising alternative due to their rapid response, potential for miniaturization, and capability for continuous monitoring, making them well-suited to support diabetes care through timely and user-friendly detection.

Glucose detection can be divided into two categories based on detection methods: invasive detection and non-invasive detection. Invasive detection is currently the main method for glucose detection, which has the advantages of high measurement accuracy, wide application, and mature technology. However, invasive detection also has shortcomings such as causing damage to the test object, usually requiring pre-treatment steps, cumbersome operation, and increased detection complexity [3,4]. Non-invasive detection can avoid the above problems. Among non-invasive detection technologies, microwave sensors have attracted the attention of researchers due to their low cost, easy integration, repeatability, and ability to achieve continuous response [5,6]. Microwave resonant sensors can real-time reflect the external dielectric properties through their resonant characteristics. Therefore, changes in the response of microwave resonant sensors can be indirectly related to changes in glucose concentration, enabling real-time monitoring of glucose concentration.

In recent years, many researchers have been exploring suitable methods to develop sensors for monitoring glucose levels in the microwave and millimeter-wave frequency ranges. Nella A et al. designed a planar Yagi-Uda antenna with a resonant frequency of 5.5 GHz, which exhibited a directional radiation pattern with a peak gain of 6.74 dB. By placing test models at different positions around the antenna and observing corresponding frequency shifts, they were able to determine the glucose concentration of the test models [7]. Hanna J et al. developed a microwave-based wearable non-invasive glucose monitoring system inspired by vascular anatomy, which provides personalized blood glucose concentration monitoring tailored to the patient’s characteristics [8]. Kiani S et al. implemented a bandpass filter using a modified split-ring resonator on a substrate-integrated waveguide cavity, thereby concentrating the electric field within a specific region of the sensor. This highly concentrated electric field enhances the sensitivity and accuracy of the sensor’s glucose monitoring function [9,10].

Buragohain A et al. reported that electro/magnetic excitation can provide higher sensitivity compared to pure electrical excitation [11]. Additionally, cross-polarized excitation generates an electric field orthogonal to that produced by parasitic capacitance, which can mitigate the impact of parasitic capacitance on the sensor’s resonant response to some extent. Meanwhile, cross-polarization enhances the penetration depth of the electric field, allowing it to interact with a larger volume of the material under test (MUT), thereby improving the sensor’s sensitivity. Therefore, the position of the slots directly determines the excitation state of the resonator and has a direct impact on its performance. The team proposed a Complementary Split-Ring Resonator (CSRR) structure in which the slots are oriented solely perpendicular to the feed line, resulting in pure electrical excitation in each ring. While increasing the number of rings in the resonator enhances the electric field strength, it also leads to a more rapid increase in size. This has resulted in resonators utilizing such designs generally having relatively large dimensions [12].

According to [13,14], electrical/magnetic excitation yields higher sensitivity than pure electrical excitation. In addition, the electric field generated by cross-polarization excitation is orthogonal to that induced by parasitic capacitance, which can mitigate the impact of parasitic capacitance on the sensor’s resonant response to a certain extent. Meanwhile, cross-polarization can enhance the electric field penetration depth, enabling more sufficient interaction with the material under test (MUT) and thus improving sensor sensitivity. Therefore, the slot position directly determines the resonator’s excitation state and its performance. Reference [15] proposed a CSRR structure with slots only perpendicular to the feeder, leading to pure electrical excitation in each ring. However, increasing the number of rings to enhance the electric field strength will cause a rapid increase in resonator size, resulting in a relatively large footprint of such designs.

To address the above issues, this study focuses on a low-cost non-invasive microwave glucose sensor based on a dual complementary split-ring resonator (DS-CSRR). By adopting an FR4 substrate and adding two U-shaped slots to the DS-CSRR, the original quality factor and electric field distribution of the resonator are optimized, thereby improving the sensitivity and accuracy of glucose concentration detection. The following sections will elaborate on the basic principle of DS-CSRR, the design and equivalent circuit of the improved resonator, as well as the glucose solution modeling, experimental testing and performance verification of the proposed sensor.

## 2. Design and Fabrication

### 2.1. Structural Design of DS-CSRR

**Figure 1:**
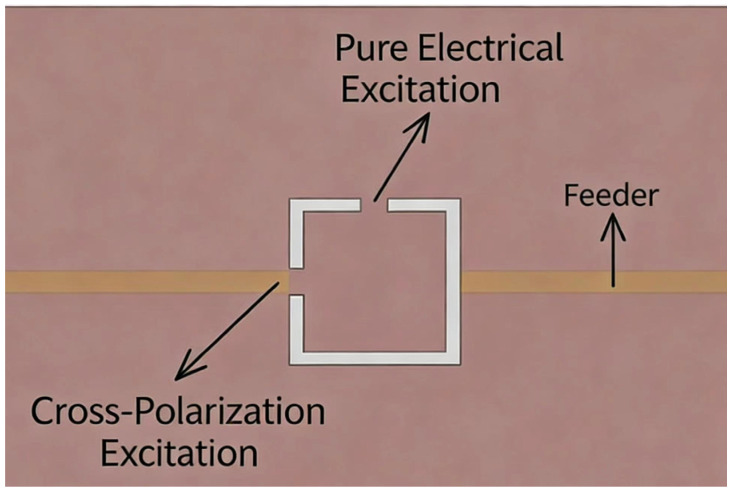
Different Excitation Modes Caused by Slit Positions.

In the design of conventional split-ring resonators (CSRRs), slits or split gaps are usually placed vertically on the feeder to provide pure electrical excitation for the resonator. However, placing CSRRs with splits along the feeder results in electrical/magnetic (cross-polarization) excitation of the resonator, as shown in Figure 1.

**Figure 2:**
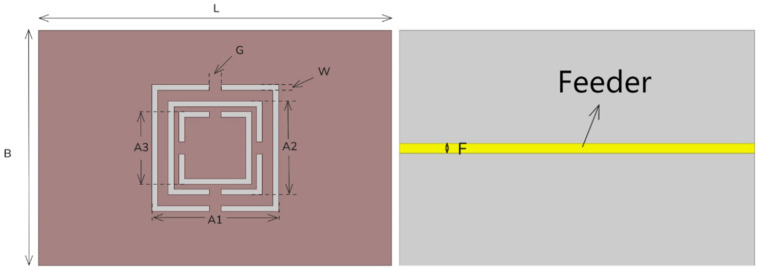
Top and Bottom Views of the Sensor.

This paper proposes a novel DS-CSRR design, as shown in Figure 2. The slots of the two inner rings are oriented along and perpendicular to the feeder, respectively, so that each ring undergoes a cross-polarization effect, achieving higher electric field strength and sensitivity than conventional CSRRs or traditional DS-CSRRs. In addition, a CSRR with pure electrical excitation is added outside the two inner rings, which can confine the cross-polarization effect between the two inner rings [16] and further enhance the electric field strength and sensitivity of the resonator.

### 2.2. Sensor Dimensions and Fabrication

The sensor’s dimensional parameters were initially derived from the fundamental principles elaborated in Section 3.1, with subsequent simulation and optimization via HFSS to finalize the structural dimensions [17,18]. The device was tailored to operate at 3.3 GHz: while higher resonant frequencies yield more pronounced variations in the dielectric constant of glucose solutions of different concentrations [19,20,21] and facilitate sensor miniaturization [22,23], the commercial FR4 substrate employed imposes practical limitations on operation above 3 GHz due to its high loss tangent (tan δ) and concomitant dielectric constant fluctuations [24], justifying the 3.3 GHz operating frequency after comprehensive trade-off analysis.

HFSS was further utilized to optimize the sensor’s structural details for an enhanced quality factor (Q) and thus improved sensitivity in glucose concentration detection. For cost-effectiveness, the sensor was fabricated on a commercially available FR4 substrate (0.8 mm thick, relative permittivity 4.4, tanδ = 0.03) with 0.035 mm-thick copper cladding on both sides. The outermost DS-CSRR structure was patterned via copper etching, with all detailed dimensional parameters listed in Table 1:

After fabricating the sensor, it is necessary to verify the fabricated sensor using liquid samples. In this experiment, a cylindrical container made of silicone rubber was used to fix the glucose solution in an appropriate position. The advantages of using silicone rubber are that this material is widely used, no special fabrication is required, and it is low-cost. In addition, it can resist corrosion, aging, and physical damage. The outer radius of the cylindrical container is 5 mm, the wall thickness is 0.5 mm, the height is 5 mm, and both the upper and lower surfaces are open. A high-viscosity polyester fiber cloth-based waterproof tape that does not affect the sensor performance was added to the substrate, and the cylindrical container was bonded on top of it as a sample container to maximize the contact between the glucose solution and the substrate.

**Figure 3:**
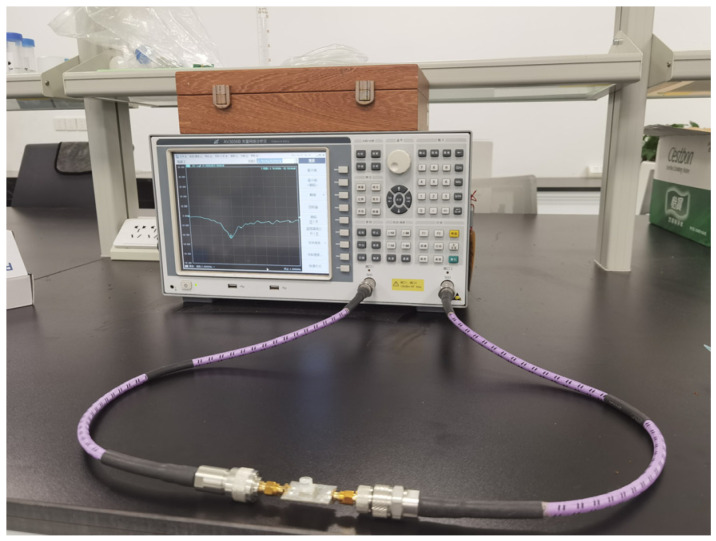
Experimental setup used for S21 parameter measurements at different glucose concentrations.

The sample container was filled with 10 μL of glucose solutions with different concentrations using a microsyringe. To eliminate the influence of ambient temperature on the actual testing of the sensor, the experiment was conducted at a constant room temperature (25 °C). The measurement process of the sensor using a Vector Network Analyzer (VNA) is shown in Figure 3. In the scanning frequency range from 2 MHz to 3.8 GHz, the S21 parameter of each liquid sample was observed and recorded.

## 3. Results and Discussion

### 3.1. Basic Principle

The split-ring resonator (SRR) structure is a quasi-static resonator, which is very suitable for measuring the complex dielectric constant of solids and liquids due to its advantages of fast response, high sensitivity, and good selectivity [13]. The SRR consists of two ends of a closed ring made of non-magnetic materials such as copper, including square rings, circular rings, etc. The current circulating around the ring generates a magnetic field, which behaves as an inductor. The gap in the middle of the ring acts as a capacitor, thus forming an LC resonant circuit. The CSRR is the complementary structure of the SRR. Based on Babinet’s principle [14], it is obtained by etching a ring structure identical to the SRR from the metal surface. Compared with the SRR, the CSRR essentially behaves as an electric dipole excited by an axial electric field. The capacitance of the SRR becomes inductance in the CSRR, and the inductance of the SRR becomes capacitance in the CSRR.

When an external material is introduced into the microwave resonant cavity, the field distribution in the cavity is perturbed due to factors such as the shape and size of the cavity, the filled medium in the cavity, and the oscillation mode in the cavity, resulting in a change in the resonant frequency of the resonant cavity. Assuming that the perturbation is small and the field distribution remains unchanged, the relationship between the resonant frequency and the change in material properties is as follows [15,20]:

(1) $$\frac{\omega -\omega_{0}}{\omega_{0}}=-\frac{\int_{V_{0}}\left[\Delta \varepsilon {\left|E_{0}\right|}^{2}+\Delta \mu {\left|H_{0}\right|}^{2}\right]dV}{\int_{V_{0}}\left[\varepsilon_{0}{\left|E_{0}\right|}^{2}+\mu_{0}{\left|H_{0}\right|}^{2}\right]dV}$$

where ω_0_ is the resonant frequency of the original cavity, ω is the resonant frequency of the cavity after perturbation, $E_{0}$ and $H_{0}$ are the field distributions without any perturbation, $\varepsilon_{0}$ and $\mu_{0}$ are the dielectric constant and permeability of the original cavity, Δε and Δμ are the changes in the dielectric constant and permeability, respectively, and v is the perturbation volume.

According to reports [21,25,26], the dielectric constant and tan δ corresponding to different glucose solution concentrations can be calculated using the Cole-Cole model to obtain the complex dielectric constant of glucose solutions with different concentrations, which is given by the following equations:

(2) $$\varepsilon_{\infty }(\chi )\approx 4.076+5.048\times 10^{-5}.\chi \;$$

(3) $$\varepsilon_{s}(\chi )\approx 80.769-0.439\times 10^{-3}.\chi$$

(4) $$\tau (\chi )\approx 8.008+0.275\times 10^{-3}.\chi$$

where $\varepsilon_{\infty }$ is the dielectric constant at infinite frequency, $\varepsilon_{s}$ is the static dielectric constant, τ is the relaxation time, and χ is the glucose concentration (unit: mg/dL, 1 mol/L = 180,000 mg/dL). Using Equations (3)–(5), the following equation can be obtained to calculate the complex dielectric constant of the glucose solution:

(5) $$\varepsilon^{*}=\varepsilon^{\prime }(\omega )-j\varepsilon^{\prime \prime }(\omega )=\varepsilon_{\infty }+\frac{\varepsilon_{\infty }-\varepsilon_{s}}{1+{\left(j\omega \tau \right)}^{1-\alpha }}+\frac{\sigma_{s}}{j\omega \varepsilon_{0}}\;$$

where ω is the angular frequency, σ is the static conductivity, $\varepsilon_{0}$ is the dielectric constant of free space, and α is the exponential parameter. When α=0, the equation is simplified to the Debye model. For materials with low conductivity, the term with static conductivity can be omitted. The relative dielectric constant $\varepsilon_{r}$ and tanδ of glucose solutions with different concentrations at 3.3 GHz used in this experiment were calculated using the above equation, as shown in Table 2:

### 3.2. Equivalent Circuit

**Figure 4:**
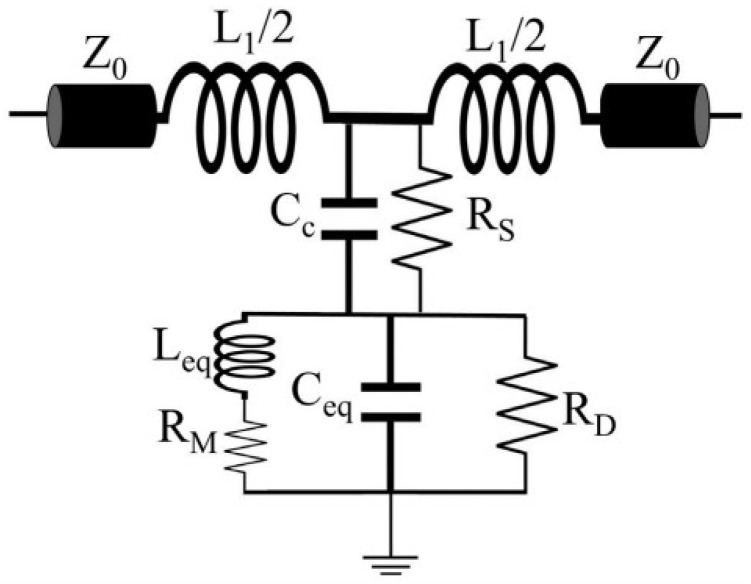
Equivalent Circuit of the Sensor.

The lumped-element equivalent circuit of the sensor is shown in Figure 4. In the circuit model, $C_{eq}$ and $L_{eq}$ are the equivalent capacitance and inductance of the CSRR. $C_{c}$ is the coupling capacitance between the microstrip line on one side and the CSRR on the other side. $L_{1}$ is the inductance of the microstrip line, $Z_{0}$ is the characteristic impedance of the microstrip line between the CSRR and the input/output ports, $R_{M}$ and $R_{D}$ are the ohmic loss and dielectric loss of the CSRR, respectively, and $R_{D}$ is also the substrate loss. The resonant frequency of this equivalent circuit is given by the following equation [18,19]:

(6) $$f_{0}=\frac{1}{2\pi \sqrt{L_{eq}\left(C_{eq}+C_{c}\right)}}$$

When the sensor is loaded with the MUT, the electric field changes, leading to an increase in the $C_{eq}$ of the sensor, thereby causing a shift in the resonant frequency of the sensor. For test objects with different dielectric constants, the resonant frequency of the sensor varies, usually decreasing as $C_{eq}$ increases. By observing the shift in the resonant frequency, the dielectric constant can be determined, which indicates the relationship between the resonant frequency and the dielectric constant of the test object.

Based on the above design concept, the sensor in this study uses three square rings as sensing elements (on the basis of the traditional single-ring CSRR). Compared with the original single-ring structure, the three-square rings increase the overall equivalent capacitance of the resonator, resulting in a relative decrease in the resonant frequency. At the same time, the electric field density between the microstrip line and the rings is significantly increased, thereby improving the quality factor (Q) of the resonator. A high Q value means improved frequency resolution, which is conducive to achieving better detection sensitivity [17]. The quality factor describes the ratio of energy stored to energy dissipated per cycle, which can be calculated as [18]:

(7) $$\;Q=2\pi \frac{E_{store}}{E_{loss}}$$

In engineering, the quality factor is also defined as the ratio of the center frequency to the −3 dB bandwidth [19]:

(8) $$Q=\frac{f_{0}}{∆f}$$

Li et al. [19] validated the design logic of improving the energy storage efficiency of passive devices and reducing parasitic losses through topological optimization via a cross-symmetric inductor structure for LC-VCOs. This design philosophy is highly consistent with the strategy adopted in this work: the introduction of U-shaped slots to realize cross-polarization excitation in the DS-CSRR also reduces the energy dissipation caused by parasitic capacitance and inductance, thereby effectively enhancing the loaded Q-factor of the resonator. Both studies confirm that structural innovation is a feasible and effective approach to optimizing the resonant characteristics of passive microwave devices by suppressing intrinsic loss.

**Figure 5:**
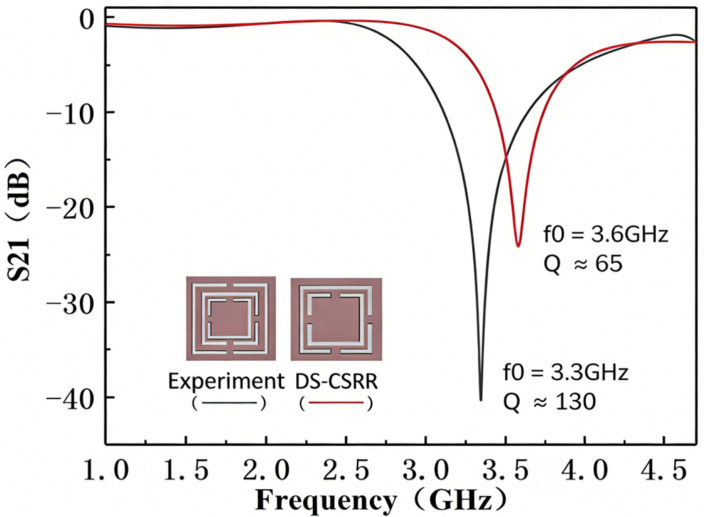
Comparison of Different CSRR Structures.

A comparison between the design in this experiment and the DS-CSRR design without the outer third ring was conducted in High Frequency Structure Simulator (HFSS), and the results are shown in Figure 5. It can be seen that under no-load conditions, the DS-CSRR proposed in this experiment has a lower resonant frequency due to the addition of two U-shaped slots. At the same time, the sharpness of the resonant frequency and the depth of the notch are significantly improved, indicating that the Q value of the resonator is enhanced and the frequency resolution is improved, enabling better performance in liquid detection. The modified Dual Complementary Split-Ring Resonator (DS-CSRR) design increases the quality factor (Q) by 130 compared with the conventional DS-CSRR.

### 3.3. Sensitivity

**Figure 6:**
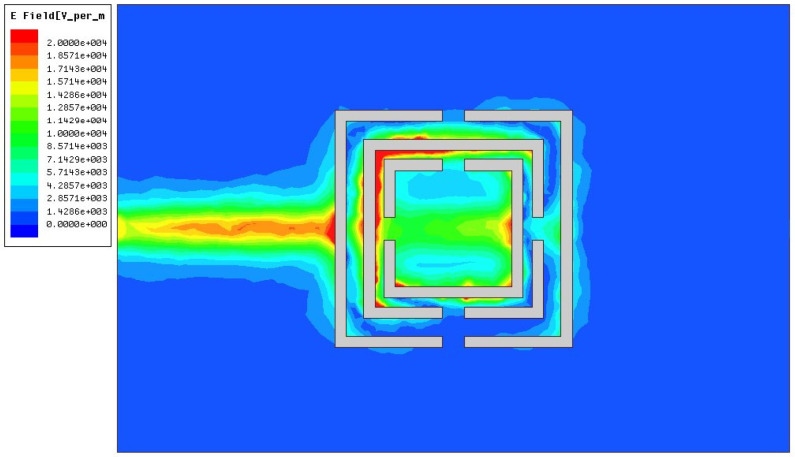
Surface Electric Field Distribution of the Sensor at 3.3 GHz.

Figure 6 shows the simulated electric field distribution on the sensor surface. It can be seen that the electric field is highly concentrated at the upper left corner under the action of the edge electric field, which means that this electric field will interact strongly with the sample in this area. Therefore, placing the sample container in the upper left corner can provide maximum sensitivity.

**Figure 7:**
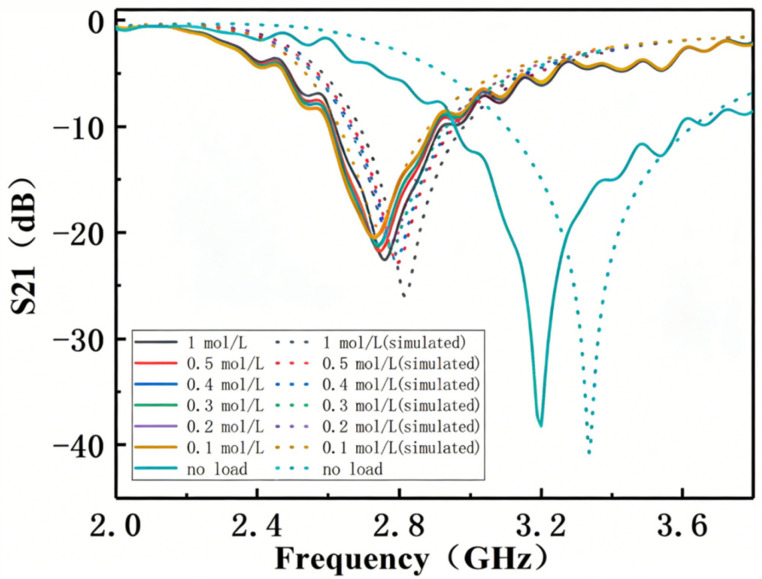
Measurement of S21 with Glucose Solutions of Different Concentrations.

In this experiment, the S21 parameter of the sensor was measured to record the resonant frequencies without any solution and with glucose solutions of different concentrations. First, the S21 of the sensor without any solution was measured to verify the consistency between the simulation and the actual situation, and the loaded quality factor (Q no-load conditions) of the resonator was determined to be 130, which was read directly from the instrument and recorded. After that, 10 μL of glucose solutions with different concentrations were added to the sample container of the sensor using a microsyringe. After each test, the sensor was cleaned and dried according to the standard experimental procedure to ensure that the notch of S21 returned to the no-load frequency position measured earlier. The experiment was conducted in a constant room temperature environment. To verify the robustness of the sensor, the experimental results obtained from the Vector Network Analyzer (VNA) were compared with the simulation results from HFSS. The final results are shown in Figure 7. It can be clearly seen that after loading the glucose solution into the sensor under no-load conditions, the resonant frequency shifts to the left. Subsequently, after loading the glucose solution, the resonant frequency shifts to the left compared with the no-load state; as the concentration of the loaded glucose solution increases, the relative dielectric constant of the solution decreases (shown in Table 2), and the resonant frequency increases slightly (shifts to the right) relative to the low-concentration glucose solution, presenting a linear frequency shift characteristic. The sensor proposed in this paper also achieves the characteristic of linear frequency shift.

For the glucose concentration sensor, sensitivity analysis can be performed from the perspective of linear regression using the following equation [27]:

(9) $$S=\frac{∆F}{∆C}$$

where ∆C is the change in glucose solution concentration, and ∆F is the relative shift in the resonant frequency.

**Figure 8:**
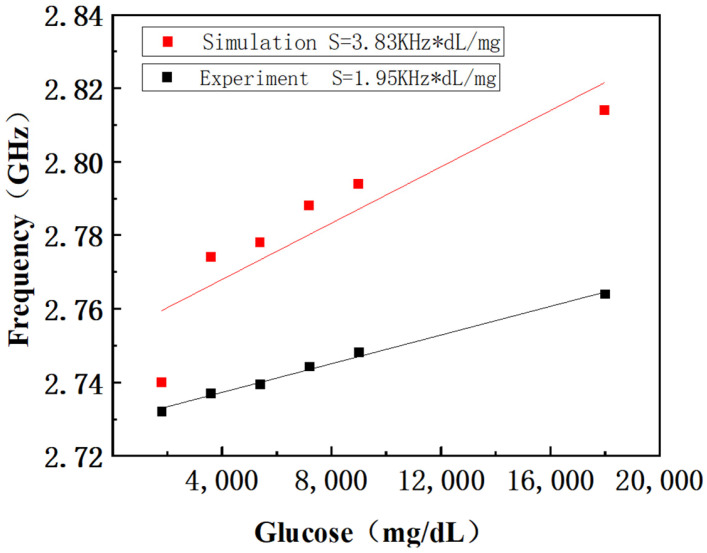
Linear fitting plot.

The resonant frequencies obtained from simulations and experiments, along with the corresponding glucose concentrations, were input into Origin 2021 software for linear fitting, as shown in Figure 8. Analysis shows that the sensitivity obtained from HFSS simulation is 3.83 kHz/(mg·dL^−1^), and the experimentally measured sensitivity is 1.95 kHz/(mg·dL^−1^), with the deviation mainly caused by manufacturing defects and material property differences.

Table 3 shows the detailed statistical parameters of linear fitting for simulation and experimental frequency responses to glucose concentration.

It is worth noting that there are some differences between the HFSS simulation results and the experimental results. This may be due to typical manufacturing defects, losses on the SMA connector, and possible differences between the material properties of the sample used in the simulation and the actual sample. In addition, the uneven thickness and overall thickness tolerance of ordinary low-grade FR4 circuit boards may also cause differences.

The performance of this sensor was compared with some existing sensors in the literature for glucose solution concentration monitoring, as shown in Table 4. Compared with other existing works in references [28,29,30,31], the sensor proposed in this paper provides a clear frequency shift value at a lower cost.

## 4. Conclusions

This study designed and fabricated a low-cost non-invasive microwave sensor for glucose concentration detection based on the DS-CSRR structure, which addresses the shortcomings of low sensitivity and large size of traditional microwave glucose sensors. The key design parameters of the sensor were optimized by HFSS simulation, with the final dimensions of 20 × 30 × 0.8 mm^3^, a resonant frequency of 3.3 GHz and a loaded Q factor of 130 under no-load conditions. By introducing two U-shaped slots into the traditional DS-CSRR, the sensor realizes cross-polarization excitation, which effectively enhances the electric field concentration and the interaction between the electric field and the detected solution, thus improving the detection sensitivity.

The sample container was placed in the high electric field concentration area of the sensor to maximize the detection sensitivity. Under the constant temperature condition of 25 °C, the S21 parameters of glucose solutions with different concentrations (0.1–1 mol/L) were measured by VNA, and the results showed a linear relationship between glucose concentration and resonant frequency shift, with the measured sensitivity reaching 1.95 kHz/(mg·dL^−1^). The consistency between the experimental results and HFSS simulation results verifies the rationality of the sensor design and the validity of the measurement results. Compared with existing microwave glucose sensors reported in the literature, the proposed sensor has the advantages of miniaturization (600 mm^2^), low cost (FR4 substrate + standard PCB fabrication), and high sensitivity, which lays a solid foundation for the development of non-invasive glucose detection technology.

However, this study still has some limitations. First, the sensor was only verified with glucose solutions in the laboratory, and its performance in actual human blood glucose detection needs to be further tested (considering the interference of human skin, blood components, etc.). Second, the measured sensitivity is lower than the simulation result due to manufacturing defects and material losses, and the fabrication process can be further optimized to reduce the deviation. In future research, we will focus on the following aspects: (1) optimizing the sensor structure to further improve the Q factor and detection sensitivity; (2) conducting in vitro and in vivo tests of the sensor, and design a wearable packaging structure to realize real-time monitoring of human blood glucose; and (3) studying the anti-interference technology of the sensor to reduce the influence of environmental factors (temperature, humidity) and human tissue interference on the detection results.

In summary, the proposed DS-CSRR-based microwave glucose sensor provides a low-cost and miniaturized solution for non-invasive glucose concentration detection, and its structural optimization strategy also provides a reference for the design of other high-sensitivity microwave biosensors.

## Figures and Tables

**Table 1:** Sensor Dimension Parameters.

Parameter	L	B	A1	A2	A3	W	G	F
Dimension (mm)	30	20	10.6	8	6.2	0.5	1	0.8

**Table 2:** $\varepsilon_{r}$ and tan δ of Glucose Solutions with Different Concentrations at 3.3 GHz.

Glucose Concentration (mol/L)	0.1	0.2	0.3	0.4	0.5	1
$\varepsilon_{r}$	77.69	76.66	75.63	74.59	73.55	68.29
tan δ	0.166	0.176	0.185	0.194	0.203	0.249

**Table 3:** Statistical Parameters of Linear Fitting.

Plot	Simulation	Experiment
Equation	y = a + b × x	y = a + b × x
Weighting	Unweighted	Unweighted
Intercept	2,752,590.16393 ± 8920.3107	2,729,492.29508 ± 690.55121
Slope	3.83242 ± 0.97503	1.94836 ± 0.07548
Sum of squared residuals	6.26308 × 10^8^	3,753,357.37705
Pearson’s r	0.89126	0.99701
R-squared	0.79434	0.99403
Adjusted R-squared	0.74292	0.99254

**Table 4:** Comparison between the Proposed Sensor and Existing Literature.

No.	Resonant Frequency (GHz)	Sensitivity kHz/(mg·dL^−1^)	Quality Factor	Size (mm^2^)
[28]	4.35	3	85	>756
[29]	4.7	2.81	N	3600
[30]	1.42	0.3	100	3140
[31]	1.156	0.1	3850	>756
This Experiment	3.3	1.95	130	600

## Data Availability

The data presented in this study are available on request from the corresponding author.
